# Anti-Inflammatory Effect of Triterpene Saponins Isolated from Blue Cohosh (*Caulophyllum thalictroides*)

**DOI:** 10.1155/2012/798192

**Published:** 2012-09-05

**Authors:** Yeonju Lee, Jae-Chul Jung, Zulfiqar Ali, Ikhlas A. Khan, Seikwan Oh

**Affiliations:** ^1^Department of Neuroscience and Tissue Injury Defense Research Center, School of Medicine, Ewha Womans University, Seoul 158-710, Republic of Korea; ^2^National Center for Natural Products Research, Thad Cochran Research Center, School of Pharmacy, The University of Mississippi, Oxford, MS 38677-1848, USA

## Abstract

Blue cohosh has been used as a medicinal herb in eastern North America. It was commonly used as traditional medicines for the treatment of menopausal symptoms, rheumatic pain, and as anti-inflammatory remedy. Particularly, extract of blue cohosh roots has been used as anti-inflammatory antipyretic in traditional medicines. In the present study, we investigated the effects of blue cohosh components on the suppressive expression of iNOS or proinflammatory cytokines after the activation of microglia with lipopolysaccharide (LPS). The expression of iNOS, TNF-**α**, IL-1**β**, and IL-6 was determined by western blotting or gene expression. Blue cohosh treatment suppressed the elevation of LPS-induced iNOS expression in a concentration-dependent manner in microglia cells. Blue cohosh constituents also suppressed the expression of TNF-**α**, IL-1**β**, and IL-6. In addition, blue cohosh extract suppressed the expression of COX-2, iNOS, and proinflammatory cytokines in adrenal glands of mice. These results demonstrate that constituents of blue cohosh exert anti-inflammatory effects through the inhibition of expression of iNOS and proinflammatory cytokines. Therefore, blue cohosh may have therapeutic potential for the treatment of inflammation-related diseases.

## 1. Introduction

The central nervous system includes two major cell types, neurons and glial cells which contain astrocytes, oligodendrocytes, and microglia [1]. Microglia play an important role as principal immune cells in the infectious, traumatic, inflammatory, ischemic, neurodegenerative, and neuroinflammatory conditions [2–5]. Microglia of the CNS is activated in response to brain injury. In diverse pathological conditions of brain, microglial activation is induced by various inflammatory mediators such as cytokine, neuronal death, and abnormal protein aggregation. While microglial activation is necessary and critical for host defense, overactivation of micoglia is neurotoxic. Thus, microglial activation plays an important role in the progression of neurodegenerative disease such as Alzheimer's disease and Parkinson disease [6]. 

Lipopolysaccharide (LPS) is representative microglial activators. The inflammatory response of activated microglia appears to be consistent although the nature of the stimuli various. Especially, LPS which is sensed by a toll-like receptor (TLR) had commonly been utilized for induction of inflammatory response [7]. LPS-stimulated microglia is known to release proinflammatory cytokines and oxidants such as TNF-*α*, IL-6, IL-1*β*, and nitric oxide [8]. Their activity is neutralized by an anti-inflammatory response that prevents excessive damage to the host. Excessive or deficient production of some cytokines can lead to disproportionate pathology or immune supression [9]. 

Thus it may be functionally important to tightly regulate that the degree of microglial activation could be a good therapeutic target to resist neurodegenerative disease. For the last decade, efforts have been made to develop anti-inflammatory agents that are able to inhibit microglial activation. However, the long-term administration of anti-inflammatory drugs is limited due to the side effects. Therefore, novel anti-inflammatory agents with fewer side effects are needed.

The involvement of the peripheral nervous system (PNS) is relatively common in some neurodegenerative proteinopathies of the brain and may be pathogenetically and diagnostically important [11]. The pathological process may target the PNS and CNS at the same time. In multiple systems, atrophy and numerous glial cytoplasmic inclusions are widely distributed in the CNS [12]. Neurofibrillary tangles can occur in the sympathetic and spinal ganglia in tauopathy although they appear to develop independently of cerebral Alzheimer's disease pathology. Peripheral ganglia and visceral organs are also involved in polyglutamine diseases [13]. Further elucidation and characterization of PNS lesions will have implications for intravital biopsy diagnosis in neurodegenerative proteinopathy, particularly in Parkinson's disease.

Papoose root (blue cohosh) is a medicinal herb in eastern North America. It was commonly used in traditional medicines as treatment for menopausal symtoms, rheumatic pain, and as anti-inflammatory remedy. Especially, extracts of the roots and rhizomes of blue cohosh have been administered in traditional medicines as anti-inflammatory and antipyretic. Blue cohosh contains a number of alkaloids and glycosides, of which the alkaloid methylcysteine and the glycoside caulosaponin (steroidal saponin) seem to contribute most of the physiological activity. Blue cohosh's oxytocic (hastening childbirth) effects are apparently produced by the glycoside caulosaponin, a derivative of the triterpenoid saponin hederagenin. These substances are considered to be phytoestrogen because of their steroid backbone. Their chemical structure is similar to that of anti-inflammatory glucocorticoids, but the contribution of these compounds to anti-inflammatory effects has yet to be investigated. In the present study, we assessed the in vitro and in vivo effects of blue cohosh on LPS-induced cytokines in BV2 cells and mice. The aim of the present study was to elucidate the anti-inflammatory properties of blue cohosh constituents in vitro and in vivo on LPS-induced NO production and anti-inflammatory cytokine expression.

## 2. Materials and Methods

### 2.1. Reagents

**Figure 1:**
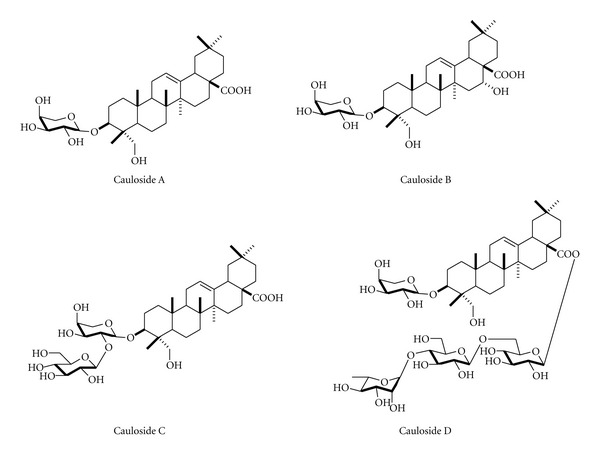
The structures of caulosides A–D from blue cohosh. Each cauloside was separated from crude saponin of blue cohosh. The % ratio of cauloside A–D to saponin was 0.18%, 0.38%, 1.93%, and 4.8%, respectively. (Data obtained from reference [10].)

LPS (*Escherichia coli*, O111:B4) was obtained from Sigma-Aldrich (St. Louis, MO, USA). Cell culture ingredients were obtained from Invitrogen (Carlsbad, CA, USA). All other reagents were obtained from Sigma-Aldrich. Roots powder (4.0 kg) of blue cohosh (*Caulophyllum thalictroides*) was extracted with MeOH at room temperature. The combined extracts were evaporated under reduced pressure to afford a brown powder (592 g). A portion (292.0 g) was dissolved in H_2_O and extracted with EtOAc. The H_2_O layer was extracted with n-butanol and evaporated to obtain a dried brown material (234 g, crude saponin). A portion of the n-butanol soluble (100 g) was resolved into fractions by column chromatography. Caulosides A–D were separated (cauloside A, 177 mg; cauloside B, 375 mg; cauloside C, 1.93 g; cauloside D, 4.8 g) and identified as triterpene saponins through column purification and NMR spectroscopy as described in detail [10]. Blue cohosh crude saponin and caulosides A–D (Figure 1) were kindly supplied from Dr. Ikhlas Khan (NCNPR, University of Mississippi, MS, USA). 

### 2.2. Animals and Agent Treatment

All the experiments were carried out using male ICR mice weighing 28–30 g purchased from the Orient Co., Ltd. (Seoul, Korea), according to the guidelines of the Animal Care and Use Guidelines of Ewha Womans University, Korea. The mice were housed 6 or 8 per cage, allowed access to water and food ad libitum, and maintained at an ambient temperature of 23°C with 40–50% humidity and a 12 h diurnal light cycle (light on 07:00-19:00). 

Seven week-old male mice were injected with saline or LPS (1 mg/kg). LPS was dissolved in saline and injected intraperitoneally. Blue cohosh crude extract (200 mg/kg) was administered orally 30 min before LPS injection. Control animals were injected with equivalent volumes of saline. 

### 2.3. Cell Culture

The murine BV2 cell line (a generous gift from W. Kim, Korea Research Institute of Bioscience and Biotechnology, Daejeon, Korea), which is immortalized after infection with a *v-raf/v-myc* recombinant retrovirus, exhibits the phenotypic and functional properties of reactive microglial cells [14]. BV2 cells were maintained at 37°C at 5% CO_2_ in Dulbecco's modified Eagle's medium (DMEM) supplemented with 10% FBS, 100 g/mL streptomycin, and 100 U/mL penicillin. BV2 cells were grown in 24-well plates at a concentration of 1 × 10^5^ cells/well followed by proper treatment.

### 2.4. Nitrite Assay

NO production from activated microglial cells was determined by measuring the amount of nitrite, a relatively stable oxidation product of NO, as described previously [15]. Cells were incubated with or without LPS in the presence or absence of various concentrations of compounds for 24 h. The nitrite accumulation in the supernatant was assessed by the Griess reaction. In brief, an aliquot of the conditioned medium 50 *μ*L was mixed with an equal volume of 1% sulfanilamide in water and 0.1% *N*-1-naphthylethylenediamine dihydrochloride in 5% phosphoric acid. The absorbance was determined at 540 nm in an automated microplate reader.

### 2.5. Immunoblot Analysis

BV2 cells were washed twice with ice-cold phosphate-buffered saline (PBS) and then lysed in ice-cold modified lysis buffer (150 mM NaCl, 50 mM Tris, 1 mM EDTA, 0.01% Triton X-100, and protease inhibitors, pH 8.0), and cellular debris was cleared by centrifugation. Samples were assayed for protein concentration using bicinchoninic acid reagents (Pierce Chemical, Rockford, IL, USA). The supernatants were aliquoted and stored at 70°C until use. Proteins were separated by SDS-polyacrylamide gel electrophoresis and transferred to a polyvinylidene difluoride membrane. The membrane was blocked with 5% skim milk in tris-buffered saline/Tween 20 solution. The blots were incubated with the antibodies of TNF-*α*, IL-6, and iNOS (Cell Signaling Technology Inc., Danvers, MA, USA). GAPDH (Santa Cruz Biotechnology, Inc., Santa Cruz, CA, USA) was performed as an internal control. After washing with tris-buffered saline/Tween 20, horseradish peroxidase-conjugated secondary antibodies (Cell Signaling Technology Inc.) were applied, and the blots were developed using the enhanced chemiluminescence detection kit (GE Healthcare, Chalfont St. Giles, Buckinghamshire, UK).

### 2.6. Polymerase Chain Reaction. 

ICR mice were stimulated by injection (i.p.) with LPS in the absence or presence of blue cohosh crude for 24 h. Total RNA was isolated from adrenal gland of ICR mice using TRIzol (Invitrogen) according to the manufacturer's instructions. For cDNA synthesis, 2 *μ*g of total RNA was reverse transcribed using the SuperScript First-Strand Synthesis System (Invitrogen). cDNA was amplified by polymerase chain reaction (PCR) using primers for COX-2 (F: AAGACTTGCCAGGCTGAACT, R: CTT CTGC AGTCCAGGTTCAA), iNOS (F: GTGTTCCACCAGGAGATGTTG, R: CTCCT GCCCACTGAGTTCGTC), TNF-*α* (F: TGTCTCAGCCTCTTCTCATT, R: GTATG AGATAGCAAATCGGC), IL-1*β* (F: AGCAACGACAAAATA CCTGT, R: CAGTC CAGCCCATACTTTAG) and IL-6 (F: CCACTTCACAAGTCG GAGGC, R: CCAG CTTATCTGTTAGGAGA). PCR products were separated by 1% agarose gel electrophoresis and visualized by ethidium bromide staining. 

### 2.7. Statistical Analysis

All values were expressed as mean ± S.E.M., and comparisons between groups were performed using analysis of variance followed by the Student-Newman-Keuls test for multiple comparisons. The results are representative of three independent experiments done in duplicate. Differences with *P* < 0.01, and *P* < 0.001 were considered as statistically significant.

## 3. Results

### 3.1. Blue Cohosh Constituents Suppressed the LPS-Induced NO Generation and iNOS Expression in Microglia. 

**Figure 2:**
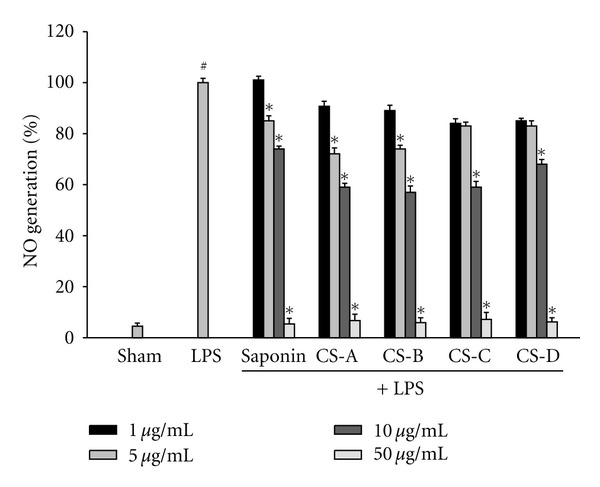
The suppression of NO generation in LPS-treated BV2 microglial cells. Cells were treated with 100 ng/mL LPS or LPS plus blue cohosh constituents for 24 h. At the end of incubation, 50 *μ*L of the medium was collected to measure nitrite production. The amount of NO in the supernatant fractions was measured by using the Griess reagent. All values are expressed as mean ± S.E.M. from three independent experiments. Data were analyzed by one-way ANOVA for multiple comparison and Student-Newman-Keuls test as post hoc test. ^#^ *P* < 0.001 in comparison with sham, *P < 0.01 in comparison with LPS.

**Figure 3:**
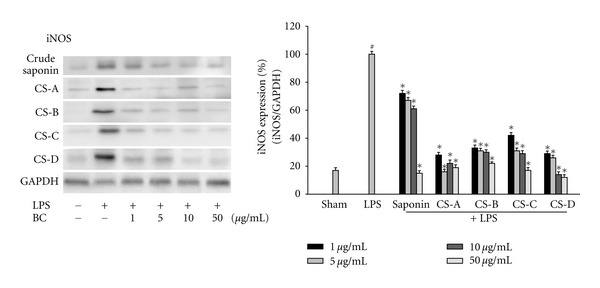
Effects of blue cohosh constituents on iNOS protein expression in LPS-treated microglial cell. BV2 microglial cells were treated by applying the blue cohosh with 100 ng/mL LPS for 24 h. The protein was extracted after 24 h of LPS treatment. iNOS protein levels were measured using western blot. Blue cohosh repressed the LPS-induced expression of iNOS protein in activated microglia. All values are expressed as mean ± S.E.M. from three independent experiments. Data were analyzed by one-way ANOVA for multiple comparison and Student-Newman-Keuls test as post hoc test. ^#^ *P* < 0.001 in comparison with sham, *P < 0.01 in comparison with LPS.

To investigate the anti-inflammatory effect of blue cohosh constituents, the LPS-induced production of NO was measured in the presence or absence of blue cohosh constituents in BV2 microglial cells. Microglial cells were cotreated with blue cohosh constituents (1–50 *μ*g/mL) and LPS (100 ng/mL) for 24 h. Most of these compounds suppressed the generation of NO in activated microglia in a dose-dependent manner (Figure 2). These findings suggest that blue cohosh active components may suppress the LPS-induced inflammatory response through inhibition of NO generation. Therefore, these results showed that blue cohosh had anti-inflammatory response. In preliminary test, crude saponin or caulosides A–D (1–50 *μ*g/mL) were exposed to BV2 cells with LPS for 24 h to determine the possibility of cytotoxicity of blue cohosh saponin. The cell viability was not modulated by the treatment of blue cohosh saponin (data not shown). The expression of iNOS was highly elevated by LPS, and this expression was inhibited by cauloside A–D or crude saponin (Figure 3). These results implied that suppression of NO generation by blue cohosh might be due to the inhibition of iNOS protein expression.

### 3.2. Blue Cohosh Constituents Reduced the LPS-Induced Expression of Proinflammatory Cytokines. 

**Figure 4:**
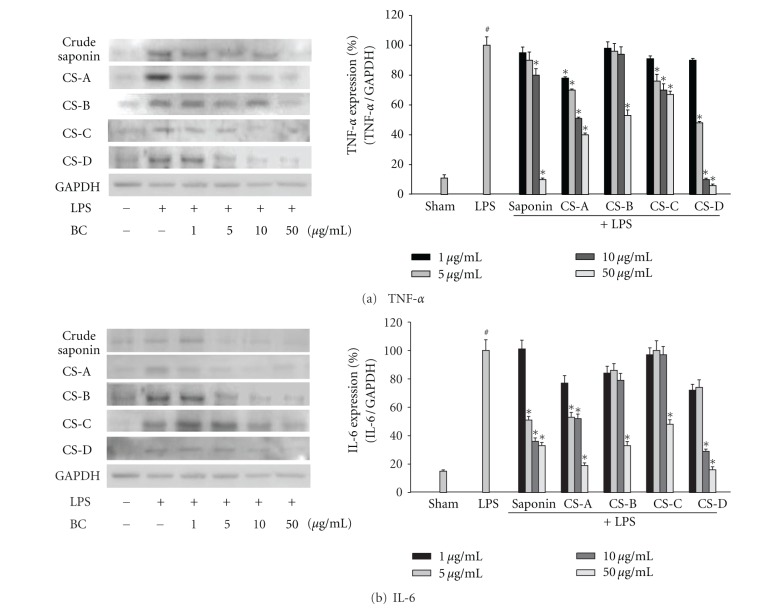
Effects of blue cohosh constituents on the LPS-induced expressions of TNF-*α* and IL-6 expression in BV2 cells. Microglial cells were treated by applying the blue cohosh constituents with 100 ng/mL LPS for 24 h. Expression of TNF-*α* and IL-6 was measured by immunoblot analysis. Blue cohosh suppressed the TNF-*α* and IL-6 in LPS-activated microglia, respectively. GAPDH was used as an internal control. Cell extracts were collected from cultured microglia after activation by LPS with cotreatment of blue cohosh, and immunoblot analysis was performed using TNF-*α* and IL-6 antibodies. Blue cohosh inhibited the activation of TNF-*α* and IL-6 at different dose. All values are expressed as mean ± S.E.M. from three independent experiments. Data were analyzed by one-way ANOVA for multiple comparison and Student-Newman-Keuls test as post hoc test. ^#^ *P* < 0.01 in comparison with sham, *P < 0.01 in comparison with LPS.

Blue cohosh exerted an anti-inflammatory effect on LPS-induced responses accompanied by the expression of proinflammatory cytokines. Primary microglial cells were cotreated with constituents of blue cohosh and LPS for 24 h. The expression levels of the proinflammatory cytokines protein, TNF-*α*, and IL-6, were reduced by treatment with blue cohosh constituents in a dose-dependent manner (Figure 4). Interestingly, cauloside A and cauloside D showed relatively strong suppressing effect on the cytokine expressions. These results indicated that blue cohosh constituents have an anti-inflammatory effect on the expression of LPS-induced proinflammatory cytokines with structure specificity in microglia.

### 3.3. Blue Cohosh Crude Saponin Reduced the LPS-Induced Elevation of Proinflammatory Cytokine Expression in Mice

**Figure 5:**
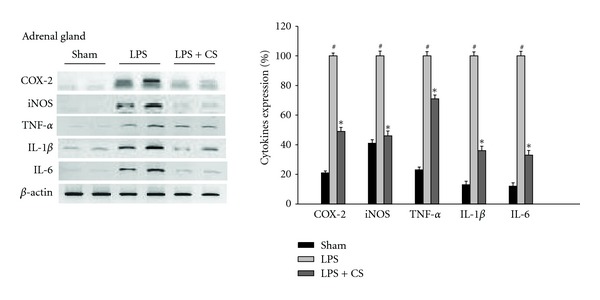
Effects of blue cohosh crude saponin on cytokines mRNA expression in LPS-treated mice. ICR mice were treated by the blue cohosh crude saponin (200 mg/kg) with 1 mg/kg LPS for 24 h. Expression of COX-2, iNOS, TNF-*α* IL-1*β*, and IL-6 was measured by PCR analysis. Blue cohosh crude suppressed the COX-2, iNOS, TNF-*α* IL-1*β*, and IL-6 in LPS-activated adrenal gland of mice respectively. *β* actin was used as an internal control. mRNA were collected from adrenal gland of ICR mice after injection of LPS with or without blue cohosh crude extract. All values are expressed as mean ± S.E.M. from three independent experiments. Data were analyzed by one-way ANOVA for multiple comparison and Student-Newman-Keuls test as post hoc test. ^#^ *P* < 0.001 in comparison with sham, *P < 0.01 in comparison with LPS.

Blue cohosh crude saponin exerted an anti-inflammatory effect on LPS-induced responses accompanied by the expression of proinflammatory cytokines in mice. Adrenal glands of ICR mice were collected after oral administration of blue cohosh crude saponin (200 mg/kg) 30 min prior to the LPS injection. Twenty-four hours after LPS injection, the mRNA expression levels of the COX-2, iNOS, TNF-*α* IL-1*β*, and IL-6 were reduced by pretreatment with blue cohosh crude saponin (Figure 5). These results indicated that blue cohosh crude saponin has an anti-inflammatory effect on the expression of LPS-induced proinflammatory cytokines in peripheral organ of mice. The lower dose of crude saponin (50 or 100 mg/kg) did not suppress the LPS-induced proinflammatory cytokine expression in adrenal gland of mice (data not shown). 

## 4. Discussion

The main purpose of this study was to determine the role of blue cohosh in LPS-induced inflammation. Microglia is the principal immune cells resident in the CNS [16]. The functional characteristics of microglia have received increasing attention, as these cells play a key role in the inflammatory reaction [17–22]. The activation of microglia has been suggested to occur during the development of neurodegenerative pathologies such as Alzheimer's and Parkinson's disease [6, 17]. Bacterial lipopolysaccharide (LPS) endotoxin has been widely used as an inflammagen to develope inflammation in the cells. It has also reported that LPS treatment induces neurotoxicity via microglial activation in mixed neuron and glia cultures [23]. 

In vivo studies also proved that activated microglia produces large amounts of reactive oxygen species (ROS), nitric oxide (NO), and proinflammatory cytokines such as TNF-*α* and interleukin-6 (IL-6), which, in turn, cause neuronal damage [3, 24–27]. Therefore, treatment with anti-inflammatory agents can appear to be a most promising option for treatment of neurodegenerative disease.

Thus in the present study, LPS was used to induce inflammation. Since nitric oxide (NO) is one of the main inflammatory mediators and plays an important role in neuroinflammatory disease, the inhibitory effect of the blue cohosh on the NO production was investigated in LPS-stimulated microglial cells. Blue cohosh constituents strongly inhibited NO production in LPS-stimulated BV2 microglial cells. Several lines of evidence have shown that the expression of iNOS, the key enzyme for NO, is upregulated in activated glia cells. The formation of the NO has been implicated in diverse biological activities [28]. The enzyme responsible for synthesizing NO from arginine, nitric oxide synthase (NOS) exists in two major forms: constitutive NOS (cNOS) and inducible NOS (iNOS). Inducible nitric-oxide synthase (iNOS) that is normally not present in resting cells is expressed in several pathophysiological conditions, and it produces large amounts of NO in response to inflammatory signals, such as cytokines and LPS [29]. While it is clear that NO plays an important role in inflammation, the pathologic processes are the pro- or anti-inflammatory properties of NO may vary according to NO concentration. And NO that is excessive damage to the host and had commonly been utilized for induction of inflammatory response of the neuronal cells in CNS. Therefore, the inhibition of NO production by iNOS may have potential therapeutic value in inflammatory-mediated disease treatment [30].

Our results showed that blue cohosh constituents inhibit the production of NO and iNOS expression in a dose-dependent manner in cells stimulated with LPS. LPS is stimulator which is responding the inflammation to generate TNF-*α*. Currently, the treatment of inflammation-mediated diseases has been studied to suppress TNF-*α* production which is released by LPS in microglia [31]. In the following studies, the inhibitory effect of blue cohosh constituents against the expression of TNF-*α* and IL-6 was investigated because these cytokines are also known as major proinflammatory mediators in the progress of inflammatory disease. In these studies, blue cohosh constituents treatment decreased the expression of TNF-*α*, which is induced by LPS in BV2 microglia cells in a dose-dependent manner. Especially TNF-*α*, which may potentiate damage to neuronal cells is a proinflammatory cytokine and costimulator which is thought to be mediated in the regulation of iNOS gene, predominantly through the mitogen-activated protein kinase (MAPK) and NF-*κ*B signaling pathway. Also ROS pathway plays an essential role as cytotoxic mediators or signaling molecule of glial cell responses to such stimuli. In our study, blue cohosh constituents markedly suppresses LPS-induced NO production as well as the expression of iNOS and proinflammatory cytokines TNF-*α* and IL-6. These inhibitors may suppress the activation of MAPK cascades, including ERK, p38 MAPK, and JNK, as well as NF-*κ*B, thereby linking ROS with LPS-induced signal transduction and transcriptional activation. Thus, further studies might be needed to identify the transcriptional levels and anti-inflammatory mechanisms of blue cohosh that are involved in the modulation of the NF-*κ*B pathways. 

Interestingly, blue cohosh constituents, caulosides A–D, significantly inhibit the LPS-induced NO generation at high concentration (50 *μ*g/mL). However, the expression of iNOS was strongly suppressed by the caulosides at low concentration (1–5 *μ*g/mL). These results suggest that NO production is not dependent solely on the expression of iNOS. LPS is stimulation factor on the production of NO as well as proinflammatory cytokines. Also cytokines affect the expression of iNOS through NF-*κ*B signals. LPS is recognized by TLR4 when it interacts with different extracellular protein LBP, CD14, and MD-2 to induce a signal cascade leading to the action of NF-*κ*B and production of proinflammatory cytokines. Caulosides A–D showed the almost same effect on the LPS-induced iNOS expression, but caulosides A and D show relatively strong effect on the TNF-*α* expression in our experiment. When the further experiment on the LPS-induced signal changes is done with variable caulosides, more detailed mechanism of caulosides would be defined. One of the possibilities of variable effect of caulosides A–D on the TNF-*α* or IL-6 expression is the solubility in water. Steroidal compound is not dissolved well in the water solution. Probably attached sugar on the steroid can enhance the solubility and absorption into cells. Interestingly, caulosides A and D have better inhibitory activity than caulosides B and C on the inflammation in our experiment. Cauloside D has three sugars on COO–, and these sugars would be detached by the intracellular esterase, and cauloside D becomes cauloside A in the cells. Cauloside B has more hydroxyl groups than cauloside A. Therefore, cauloside B is more polar and less soluble in water.

Immune cytokines are rapidly induced in response to tissue injury, infection, or inflammation. Cytokines act in the brain through one or more of the mechanisms. It is unclear which of these nonmutually exclusive mechanisms are involved in specific pathophysiology of the neuroimmune interface [32]. The proinflammatory cytokines have emerged on the basis of their peripheral action. Cytokines and their receptors are expressed within anterior pituitary cells. During inflammatory stress, cytokines that were stimulated with LPS are produced peripherally as well as within the hypothalamus and pituitary. Pituitary cytokine expression and action has been extensively reviewed [33, 34]. Regarding the anti-inflammatory role of blue cohosh, we hypothesized that the blue cohosh decreased the proinflammatory cytokine expression in adrenal gland of mice. This idea is in line with animal and human data showing that disruption or attenuation of adrenal gland function is linked to an increased risk for chronic inflammatory disease [35–38]. In the present study, blue cohosh saponin significantly decreased proinflammatory cytokine responses in adrenal gland. The underlying mechanisms of reduced adrenal gland function during active inflammation are unclear. In conclusion, blue cohosh constituents lead to suppressing NO production, iNOS expression, and proinflammatory cytokine expression in a dose-dependent manner. These results suggest that blue cohosh could be used as anti-inflammatory agent. 

## References

[1] Murabe Y, Sano Y (1982). Morphological studies on neuroglia. VI. Postnatal development of microglial cells. *Cell and Tissue Research*.

[2] Barone FC, Feuerstein GZ (1999). Inflammatory mediators and stroke: new opportunities for novel therapeutics. *Journal of Cerebral Blood Flow and Metabolism*.

[3] González-Scarano F, Baltuch G (1999). Microglia as mediators of inflammatory and degenerative diseases. *Annual Review of Neuroscience*.

[4] Minagar A, Shapshak P, Fujimura R, Ownby R, Heyes M, Eisdorfer C (2002). The role of macrophage/microglia and astrocytes in the pathogenesis of three neurologic disorders: HIV-associated dementia, Alzheimer disease, and multiple sclerosis. *Journal of the Neurological Sciences*.

[5] Wyss-Coray T, Mucke L (2002). Inflammation in neurodegenerative disease—a double-edged sword. *Neuron*.

[6] McGeer PL, Itagaki S, Boyes BE, McGeer EG (1988). Reactive microglia are positive for HLA-DR in the substantia nigra of Parkinson’s and Alzheimer’s disease brains. *Neurology*.

[7] Rock RB, Gekker G, Hu S (2004). Role of microglia in central nervous system infections. *Clinical Microbiology Reviews*.

[8] Barone FC, Arvin B, White RF (1997). Tumor necrosis factor-*α*: a mediator of focal ischemic brain injury. *Stroke*.

[9] Fiorentino DF, Zlotnik A, Mosmann TR, Howard M, O’Garra A (1991). IL-10 inhibits cytokine production by activated macrophages. *Journal of Immunology*.

[10] Ali Z, Khan IA (2008). Alkaloids and saponins from blue cohosh. *Phytochemistry*.

[11] Kovacs GG, Botond G, Budka H (2010). Protein coding of neurodegenerative dementias: the neuropathological basis of biomarker diagnostics. *Acta Neuropathologica*.

[12] Braak H, Del Tredici K (2008). Invited article: nervous system pathology in sporadic Parkinson disease. *Neurology*.

[13] Djaldetti R, Lev N, Melamed E (2009). Lesions outside the CNS in Parkinson’s disease. *Movement Disorders*.

[14] Bocchini V, Mazzolla R, Barluzzi R, Blasi E, Sick P, Kettenmann H (1992). An immortalized cell line expresses properties of activated microglial cells. *Journal of Neuroscience Research*.

[15] Green LC, Wagner DA, Glogowski J (1982). Analysis of nitrate, nitrite, and [15N]nitrate in biological fluids. *Analytical Biochemistry*.

[16] Thomas WE (1992). Brain macrophages: evaluation of microglia and their functions. *Brain Research Reviews*.

[17] Dickson DW, Lee SC, Mattiace LA, Yen SH, Brosnan C (1993). Microglia and cytokines in neurological disease, with special reference to AIDS and Alzheimer’s disease. *Glia*.

[18] Kreutzberg GW (1996). Microglia: a sensor for pathological events in the CNS. *Trends in Neurosciences*.

[19] Hauss-Wegrzyniak B, Dobrzanski P, Stoehr JD, Wenk GL (1998). Chronic neuroinflammation in rats reproduces components of the neurobiology of Alzheimer’s disease. *Brain Research*.

[20] Aloisi F (2000). The role of microglia and astrocytes in CNS immune surveillance and immunopathology. *Advances in Experimental Medicine and Biology*.

[21] Hirsch EC (2000). Glial cells and Parkinson’s disease. *Journal of Neurology*.

[22] Streit WJ (2000). Microglial response to brain injury: a brief synopsis. *Toxicologic Pathology*.

[23] Liu B, Wang K, Gao HM, Mandavilli B, Wang JY, Hong JS (2001). Molecular consequences of activated microglia in the brain: overactivation induces apoptosis. *Journal of Neurochemistry*.

[24] Boje KM, Arora PK (1992). Microglial-produced nitric oxide and reactive nitrogen oxides mediate neuronal cell death. *Brain Research*.

[25] Bronstein DM, Perez-Otano I, Sun V (1995). Glia-dependent neurotoxicity and neuroprotection in mesencephalic cultures. *Brain Research*.

[26] Minghetti L, Levi G (1998). Microglia as effector cells in brain damage and repair: focus on prostanoids and nitric oxide. *Progress in Neurobiology*.

[27] Liu B, Gao HM, Wang JY, Jeohn GH, Cooper CL, Hong JS (2002). Role of nitric oxide in inflammation-mediated neurodegeneration. *Annals of the New York Academy of Sciences*.

[28] Palmer RMJ, Ashton DS, Moncada S (1988). Vascular endothelial cells synthesize nitric oxide from L-arginine. *Nature*.

[29] Nathan C (1992). Nitric oxide as a secretory product of mammalian cells. *The FASEB Journal*.

[30] Hewett SJ, Csernansky CA, Choi DW (1994). Selective potentiation of NMDA-induced neuronal injury following induction of astrocytic iNOS. *Neuron*.

[31] Pawate S, Shen Q, Fan F, Bhat NR (2004). Redox regulation of glial inflammatory response to lipopolysaccharide and interferon*γ*. *Journal of Neuroscience Research*.

[32] Licinio J, Wong ML (1999). The role of inflammatory mediators in the biology of major depression: central nervous system cytokines modulate the biological substrate of depressive symptoms, regulate stress-responsive systems, and contribute to neurotoxicity and neuroprotection. *Molecular Psychiatry*.

[33] Arzt E (2001). gp130 cytokine signaling in the pituitary gland: a paradigm for cytokine-neuro-endocrine pathways. *Journal of Clinical Investigation*.

[34] Ray D, Melmed S (1997). Pituitary cytokine and growth factor expression and action. *Endocrine Reviews*.

[35] Buske-Kirschbaum A, Geiben A, Höllig H, Morschhäuser E, Hellhammer D (2002). Altered responsiveness of the hypothalamus-pituitary-adrenal axis and the sympathetic adrenomedullary system to stress in patients with atopic dermatitis. *Journal of Clinical Endocrinology and Metabolism*.

[36] Elenkov IJ, Chrousos GP (2002). Stress hormones, proinflammatory and antiinflammatory cytokines, and autoimmunity. *Annals of the New York Academy of Sciences*.

[37] Eskandari F, Sternberg EM (2002). Neural-immune interactions in health and disease. *Annals of the New York Academy of Sciences*.

[38] Heesen C, Gold SM, Huitinga I, Reul JMHM (2007). Stress and hypothalamic-pituitary-adrenal axis function in experimental autoimmune encephalomyelitis and multiple sclerosis—a review. *Psychoneuroendocrinology*.

